# 1,4-Diazo­niabi­cyclo­[2.2.2]octane tetra­chlorido­cadmate(II) monohydrate

**DOI:** 10.1107/S1600536814007533

**Published:** 2014-04-12

**Authors:** Tarek Ben Rhaiem, Habib Boughzala

**Affiliations:** a Laboratoire de Matériaux et Cristallochimie, Faculté des Sciences de Tunis, Université de Tunis El Manar, 2092 Manar II Tunis, Tunisia

## Abstract

The asymmetric unit of the title compound (C_6_H_14_N_2_)[CdCl_4_]·H_2_O contained one 1,4-di­aza­bicyclo­[2.2.2]octane dication, a tetrahedral CdCl_4_^2−^ anion and a lattice water mol­ecule. In the crystal, the solvate water mol­ecule inter­acts with the cationic and anionic species *via* N—H⋯O and O—H⋯Cl [O⋯Cl = 3.289 (7) Å] hydrogen-bond inter­actions, respectively, leading to a layered supramolecular structure extending parallel to (011).

## Related literature   

For background to this class of compounds, see: Wei & Willett (2002 ▶); Billing & Lemmerer (2009 ▶); Samet *et al.* (2010 ▶) Lemmerer & Billing (2012 ▶); Ben Rhaiem *et al.* (2013 ▶). For related structures, see: Sun & Qu (2005 ▶); Zhang & Zhu (2012 ▶).
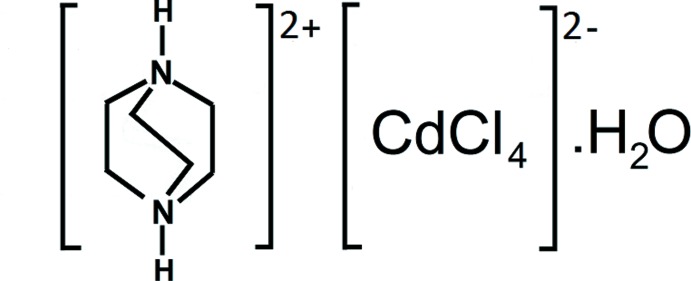


## Experimental   

### 

#### Crystal data  

(C_6_H_14_N_2_)[CdCl_4_]·H_2_O
*M* *_r_* = 386.40
Orthorhombic, 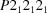

*a* = 8.528 (5) Å
*b* = 11.653 (2) Å
*c* = 13.114 (6) Å
*V* = 1303.2 (10) Å^3^
*Z* = 4
Mo *K*α radiation
μ = 2.47 mm^−1^
*T* = 298 K
0.54 × 0.43 × 0.29 mm

#### Data collection  

Enraf–Nonius CAD-4 diffractometer
Absorption correction: ψ scan (North *et al.*, 1968 ▶) *T*_min_ = 0.283, *T*_max_ = 0.536
5639 measured reflections
2837 independent reflections
2632 reflections with *I* > 2σ(*I*)
*R*_int_ = 0.075
2 standard reflections every 120 min intensity decay: 1%

#### Refinement  

*R*[*F* ^2^ > 2σ(*F* ^2^)] = 0.048
*wR*(*F* ^2^) = 0.134
*S* = 1.19
2837 reflections
135 parameters
10 restraints
H-atom parameters not refined
Δρ_max_ = 1.58 e Å^−3^
Δρ_min_ = −1.44 e Å^−3^

### 

Data collection: *CAD-4 EXPRESS* (Duisenberg, 1992 ▶); cell refinement: *CAD-4 EXPRESS*; data reduction: *XCAD4* (Harms & Wocadlo, 1995 ▶); program(s) used to solve structure: *SHELXS97* (Sheldrick, 2008 ▶); program(s) used to refine structure: *SHELXL97*; molecular graphics: *DIAMOND* (Brandenburg, 2006 ▶); software used to prepare material for publication: *publCIF* (Westrip, 2010 ▶).

## Supplementary Material

Crystal structure: contains datablock(s) I, New_Global_Publ_Block. DOI: 10.1107/S1600536814007533/ds2238sup1.cif

Structure factors: contains datablock(s) I. DOI: 10.1107/S1600536814007533/ds2238Isup2.hkl

CCDC reference: 967916

Additional supporting information:  crystallographic information; 3D view; checkCIF report

## Figures and Tables

**Table 1:** Hydrogen-bond geometry (Å, °)

*D*—H⋯*A*	*D*—H	H⋯*A*	*D*⋯*A*	*D*—H⋯*A*
N2—H2⋯O^i^	0.84	2.01	2.783 (1)	151

Symmetry code: (i) 
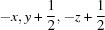
.

**Fig. 1.**
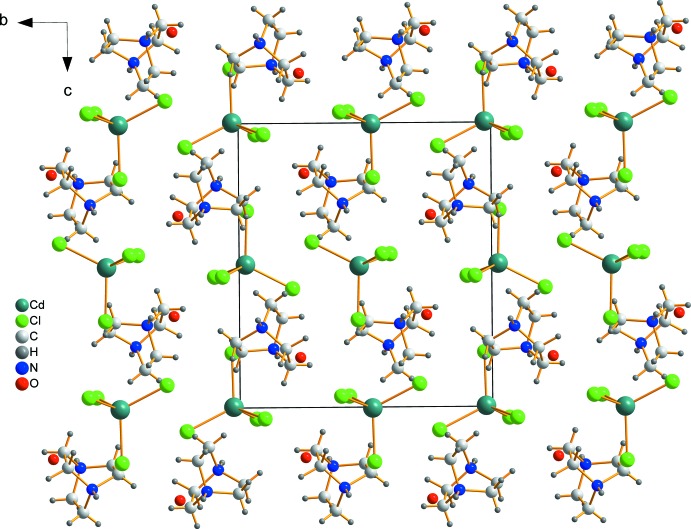
Packing diagram of (I), projected along the a axis.

**Fig. 2.**
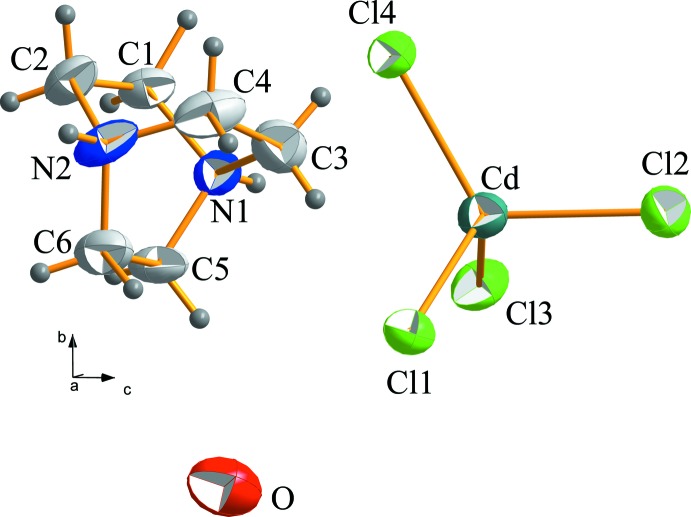
The asymmetric unit of (I), showing the atom numbering scheme. Displacement ellipsoids are drawn at 50% probability level and H atoms are shown as small spheres of arbitrary radii.

**Fig. 3.**
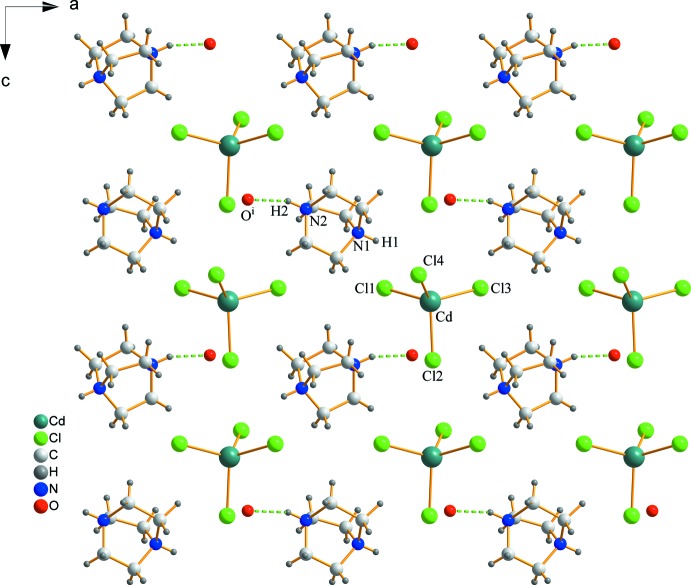
The arrangement of ions of (I), projected along the b axis. [Symmetry code: (i) -x, y+1/2, -z + 1/2.]

## supplementary crystallographic information

### 1. Comment

In recent years, a significant number of organic–inorganic hybrid materials based on metal halide units have been prepared and studied (Lemmerer & Billing, 2012). It has been shown that their structures can vary considerably, ranging from systems based on isolated polyhydra to ones containing extended chains and up to two- or three-dimensional networks (Ben Rhaiem *et al.*, 2013; Samet *et al.*, 2010; Billing & Lemmerer, 2009). Generally, the organic cations contain ammonium groups linked to the anionic framework by hydrogen bonds *via* halogenous tetrahedral vertices (Sun & Qu, 2005) and (Zhang & Zhu, 2012). In pseudopolymorphic cases, the water molecules can be able to coordinate the charged components strengthening the crystal cohesion as it was observed in (dabcoH_2_)CuCl_4_ and (dabcoH_2_)CuCl_4_·H_2_O (Wei & Willett, 2002).

The new chloridocadmate(II) compound, (C_6_H_14_N_2_) [CdCl_4_]·H_2_O (I), is self-assembled into alternating organic and inorganic layered structure. the organic part is made up of 1,4-diazabicyclo[2.2.2]octane cations and water molecules. The inorganic component contains isolated [CdCl_4_]^2-^ units. The layers are stacked along the *c* axis, as illustrated in Fig. 1.

The asymmetric unit of (I) comprises one 1,4-diazabicyclo[2.2.2]octane cation, one [CdCl_4_]^2-^ anion and a lattice occluded water molecule (Fig. 2).

The [CdCl_4_]^2-^ unit possesses a configuration of distorted tetrahedron, so that the central cadmium (II) ion is surrounded by four chlorine atoms. The Cd–Cl bond lengths vary from 2.430 (2) Å to 2.4864 (17) Å and the Cl–Cd–Cl angles fall in the range 101.80 (6)°–116.95 (6)°.

The protonated N2 atom of the organic cation interacts *via* a simple hydrogen bond with oxygen atom of the water molecule (Fig. 3 and Tab. 1).

### 2. Experimental

The title compound (C_6_H_14_N_2_) [CdCl_4_]·H_2_O, (I), was obtained by the reaction of cadmium iodide CdI_2_ (0.19 g, 0.5 mmol) with DABCO (1,4-diazabicyclo[2.2.2]octane) (0.112 g, 1 mmol) in aqueous hydrochloric acid solution with pH ranging between 3 and 4. The mixture was stirred for several minutes. Colorless crystals suitable for X-ray diffraction analysis were obtained by slow evaporation at room temperature over 2 weeks.

### 3. Refinement

Hydrogen water molecules are omited. The C—H and N—H hydrogen atoms positions are generated geometrically by HFIX *SHELXL* command.

### Crystal data

**Table:**

(C_6_H_14_N_2_)[CdCl_4_]·H_2_O	*F*(000) = 752
*M**_r_* = 386.40	*D*_x_ = 1.959 Mg m^−^^3^
Orthorhombic, *P*2_1_2_1_2_1_	Mo *K*α radiation, λ = 0.71073 Å
Hall symbol: P 2ac 2ab	Cell parameters from 2837 reflections
*a* = 8.528 (5) Å	θ = 2.4–27°
*b* = 11.653 (2) Å	µ = 2.47 mm^−^^1^
*c* = 13.114 (6) Å	*T* = 298 K
*V* = 1303.2 (10) Å^3^	Prism, colorless
*Z* = 4	0.54 × 0.43 × 0.29 mm

### Data collection

**Table:**

Enraf–Nonius CAD-4 diffractometer	2632 reflections with *I* > 2σ(*I*)
Radiation source: fine-focus sealed tube	*R*_int_ = 0.075
Graphite monochromator	θ_max_ = 27.0°, θ_min_ = 2.3°
non–profiled ω/2θ scans	*h* = −10→6
Absorption correction: ψ scan (North *et al.* (1968)	*k* = −14→1
*T*_min_ = 0.283, *T*_max_ = 0.536	*l* = −16→16
5639 measured reflections	2 standard reflections every 120 min
2837 independent reflections	intensity decay: 1%

### Refinement

**Table:**

Refinement on *F*^2^	Primary atom site location: structure-invariant direct methods
Least-squares matrix: full	Secondary atom site location: difference Fourier map
*R*[*F*^2^ > 2σ(*F*^2^)] = 0.048	Hydrogen site location: inferred from neighbouring sites
*wR*(*F*^2^) = 0.134	H-atom parameters not refined
*S* = 1.19	*w* = 1/[σ^2^(*F*_o_^2^) + (0.0587*P*)^2^ + 1.6133*P*] where *P* = (*F*_o_^2^ + 2*F*_c_^2^)/3
2837 reflections	(Δ/σ)_max_ = 0.001
135 parameters	Δρ_max_ = 1.58 e Å^−^^3^
10 restraints	Δρ_min_ = −1.44 e Å^−^^3^

### Special details

**Table:**

Experimental. Number of psi-scan sets used was 5 Theta correction was applied. Averaged transmission function was used. No Fourier smoothing was applied.
Geometry. All e.s.d.'s (except the e.s.d. in the dihedral angle between two l.s. planes) are estimated using the full covariance matrix. The cell e.s.d.'s are taken into account individually in the estimation of e.s.d.'s in distances, angles and torsion angles; correlations between e.s.d.'s in cell parameters are only used when they are defined by crystal symmetry. An approximate (isotropic) treatment of cell e.s.d.'s is used for estimating e.s.d.'s involving l.s. planes.
Refinement. Refinement of *F*^2^ against ALL reflections. The weighted *R*-factor *wR* and goodness of fit *S* are based on *F*^2^, conventional *R*-factors *R* are based on *F*, with *F* set to zero for negative *F*^2^. The threshold expression of *F*^2^ > σ(*F*^2^) is used only for calculating *R*-factors(gt) *etc*. and is not relevant to the choice of reflections for refinement. *R*-factors based on *F*^2^ are statistically about twice as large as those based on *F*, and *R*- factors based on ALL data will be even larger.

### Fractional atomic coordinates and isotropic or equivalent isotropic displacement parameters (Å^2^)

**Table:**

	*x*	*y*	*z*	*U*_iso_*/*U*_eq_	
Cd	0.74636 (5)	0.52500 (4)	0.50315 (3)	0.04606 (17)	
Cl1	0.52000 (18)	0.40196 (13)	0.46103 (13)	0.0465 (3)	
Cl2	0.7583 (2)	0.53068 (14)	0.69230 (11)	0.0544 (4)	
Cl3	0.9940 (2)	0.43346 (15)	0.46078 (15)	0.0557 (4)	
Cl4	0.6884 (2)	0.71103 (15)	0.41831 (12)	0.0542 (4)	
C1	0.4091 (7)	0.6836 (5)	0.2060 (5)	0.0457 (13)	
H1A	0.498 (5)	0.6620 (12)	0.153 (3)	0.055*	
H1B	0.4419 (18)	0.761 (4)	0.2435 (19)	0.055*	
C2	0.2540 (8)	0.6998 (6)	0.1512 (5)	0.0514 (13)	
H2A	0.2282 (17)	0.776 (5)	0.1496 (5)	0.062*	
H2B	0.2617 (9)	0.6741 (16)	0.086 (4)	0.062*	
C3	0.2824 (8)	0.6248 (7)	0.3636 (6)	0.0583 (18)	
H3A	0.3266 (11)	0.6796 (11)	0.4005 (8)	0.070*	
H3B	0.2613 (9)	0.5660 (12)	0.4042 (9)	0.070*	
C4	0.1319 (9)	0.6690 (6)	0.3149 (6)	0.065 (2)	
H4A	0.0481 (16)	0.6394 (8)	0.3470 (8)	0.078*	
H4B	0.1274 (9)	0.7458 (14)	0.3198 (7)	0.078*	
C5	0.3284 (9)	0.4857 (5)	0.2294 (6)	0.0519 (16)	
H5A	0.3330 (9)	0.4244 (11)	0.2713 (9)	0.062*	
H5B	0.3874 (13)	0.4708 (6)	0.1738 (11)	0.062*	
C6	0.1623 (9)	0.5077 (6)	0.1985 (7)	0.0586 (18)	
H6A	0.0970 (14)	0.4692 (9)	0.2392 (9)	0.070*	
H6B	0.1468 (9)	0.4843 (7)	0.1344 (12)	0.070*	
N1	0.3888 (6)	0.5887 (5)	0.2823 (4)	0.0431 (11)	
H1	0.477 (2)	0.5730 (6)	0.3082 (7)	0.052*	
N2	0.1318 (6)	0.6341 (5)	0.2067 (5)	0.0554 (15)	
H2	0.043 (2)	0.6488 (6)	0.1811 (8)	0.067*	
O	0.1563 (8)	0.2409 (6)	0.3238 (6)	0.0861 (19)	

### Atomic displacement parameters (Å^2^)

**Table:**

	*U*^11^	*U*^22^	*U*^33^	*U*^12^	*U*^13^	*U*^23^
Cd	0.0463 (3)	0.0409 (2)	0.0510 (3)	0.00213 (18)	−0.0028 (2)	0.00245 (17)
Cl1	0.0486 (8)	0.0402 (7)	0.0508 (8)	−0.0018 (6)	0.0024 (6)	−0.0039 (6)
Cl2	0.0613 (8)	0.0530 (8)	0.0490 (7)	0.0058 (9)	−0.0041 (8)	−0.0042 (6)
Cl3	0.0519 (8)	0.0515 (8)	0.0635 (9)	0.0105 (7)	0.0063 (7)	0.0105 (8)
Cl4	0.0684 (9)	0.0423 (7)	0.0520 (8)	0.0014 (7)	−0.0163 (7)	0.0047 (7)
C1	0.042 (3)	0.034 (3)	0.061 (3)	−0.005 (2)	0.004 (3)	0.007 (3)
C2	0.049 (3)	0.048 (3)	0.057 (3)	−0.006 (3)	−0.001 (3)	0.020 (3)
C3	0.063 (5)	0.057 (4)	0.056 (3)	−0.006 (3)	0.006 (3)	−0.005 (3)
C4	0.062 (4)	0.046 (4)	0.087 (5)	0.012 (3)	0.032 (4)	0.012 (4)
C5	0.055 (4)	0.026 (2)	0.075 (4)	0.001 (3)	−0.008 (3)	0.002 (3)
C6	0.061 (4)	0.041 (3)	0.075 (5)	−0.008 (3)	−0.016 (4)	0.006 (3)
N1	0.042 (2)	0.040 (2)	0.047 (3)	0.002 (2)	−0.005 (2)	0.004 (2)
N2	0.038 (3)	0.044 (3)	0.084 (4)	0.004 (2)	−0.004 (3)	0.027 (3)
O	0.085 (4)	0.070 (4)	0.103 (5)	−0.014 (3)	0.008 (4)	−0.009 (4)

### Geometric parameters (Å, º)

**Table:**

Cd—Cl3	2.430 (2)	C3—H3B	0.8860
Cd—Cl1	2.4673 (18)	C4—N2	1.476 (11)
Cd—Cl2	2.4835 (19)	C4—H4A	0.8977
Cd—Cl4	2.4864 (17)	C4—H4B	0.8977
C1—N1	1.502 (8)	C5—N1	1.479 (9)
C1—C2	1.517 (9)	C5—C6	1.496 (10)
C1—H1A	1.0614	C5—H5A	0.9026
C1—H1B	1.0614	C5—H5B	0.9026
C2—N2	1.485 (8)	C6—N2	1.499 (9)
C2—H2A	0.9127	C6—H6A	0.8931
C2—H2B	0.9127	C6—H6B	0.8931
C3—N1	1.461 (9)	N1—H1	0.8477
C3—C4	1.524 (10)	N2—H2	0.8420
C3—H3A	0.8860		
			
Cl3—Cd—Cl1	111.93 (7)	N2—C4—H4B	110.1
Cl3—Cd—Cl2	101.80 (6)	C3—C4—H4B	110.1
Cl1—Cd—Cl2	105.73 (6)	H4A—C4—H4B	108.4
Cl3—Cd—Cl4	116.95 (6)	N1—C5—C6	108.5 (5)
Cl1—Cd—Cl4	104.53 (6)	N1—C5—H5A	110.0
Cl2—Cd—Cl4	115.58 (6)	C6—C5—H5A	110.0
N1—C1—C2	107.9 (5)	N1—C5—H5B	110.0
N1—C1—H1A	110.1	C6—C5—H5B	110.0
C2—C1—H1A	110.1	H5A—C5—H5B	108.4
N1—C1—H1B	110.1	C5—C6—N2	108.3 (5)
C2—C1—H1B	110.1	C5—C6—H6A	110.0
H1A—C1—H1B	108.4	N2—C6—H6A	110.0
N2—C2—C1	108.4 (5)	C5—C6—H6B	110.0
N2—C2—H2A	110.0	N2—C6—H6B	110.0
C1—C2—H2A	110.0	H6A—C6—H6B	108.4
N2—C2—H2B	110.0	C3—N1—C5	111.1 (6)
C1—C2—H2B	110.0	C3—N1—C1	110.2 (5)
H2A—C2—H2B	108.4	C5—N1—C1	109.0 (5)
N1—C3—C4	108.4 (6)	C3—N1—H1	108.8
N1—C3—H3A	110.0	C5—N1—H1	108.8
C4—C3—H3A	110.0	C1—N1—H1	108.8
N1—C3—H3B	110.0	C4—N2—C2	109.2 (6)
C4—C3—H3B	110.0	C4—N2—C6	109.9 (6)
H3A—C3—H3B	108.4	C2—N2—C6	110.5 (6)
N2—C4—C3	108.0 (5)	C4—N2—H2	109.1
N2—C4—H4A	110.1	C2—N2—H2	109.1
C3—C4—H4A	110.1	C6—N2—H2	109.1

### Hydrogen-bond geometry (Å, º)

**Table:**

*D*—H···*A*	*D*—H	H···*A*	*D*···*A*	*D*—H···*A*
N2—H2···O^i^	0.84	2.01	2.783 (1)	151

Symmetry code: (i) −*x*, *y*+1/2, −*z*+1/2.
